# The Health Effects of a Forest Environment on Subclinical Cardiovascular Disease and Heath-Related Quality of Life

**DOI:** 10.1371/journal.pone.0103231

**Published:** 2014-07-28

**Authors:** Tsung-Ming Tsao, Ming-Jer Tsai, Ya-Nan Wang, Heng-Lun Lin, Chang-Fu Wu, Jing-Shiang Hwang, Sandy-H.J. Hsu, Hsing Chao, Kai-Jen Chuang, Charles- CK. Chou, Ta-Chen Su

**Affiliations:** 1 The Experimental Forest, National Taiwan University, Nantou, Taiwan; 2 The School of Forestry and Resource Conservation, National Taiwan University, Taipei, Taiwan; 3 Department of Internal Medicine and Cardiovascular Center, National Taiwan University Hospital, Taipei, Taiwan; 4 Department of Public Health, National Taiwan University, Taipei, Taiwan; 5 Institute of Statistical Science, Academia Sinica, Taipei, Taiwan; 6 Laboratory Medicine, National Taiwan University Hospital, Taipei, Taiwan; 7 School of Public Health, Taipei Medical University, Taipei, Taiwan; 8 Research Center for Environmental Changes, Academia Sinica, Taipei, Taiwan; Supportive care, Early DIagnosis and Advanced disease (SEDA) research group, United Kingdom

## Abstract

**Background:**

Assessment of health effects of a forest environment is an important emerging area of public health and environmental sciences.

**Purpose:**

To demonstrate the long-term health effects of living in a forest environment on subclinical cardiovascular diseases (CVDs) and health-related quality of life (HRQOL) compared with that in an urban environment.

**Materials and Methods:**

This study included the detailed health examination and questionnaire assessment of 107 forest staff members (FSM) and 114 urban staff members (USM) to investigate the long-term health effects of a forest environment. Air quality monitoring between the forest and urban environments was compared. In addition, work-related factors and HRQOL were evaluated.

**Results:**

Levels of total cholesterol, low-density lipoprotein cholesterol, and fasting glucose in the USM group were significantly higher than those in the FSM group. Furthermore, a significantly higher intima-media thickness of the internal carotid artery was found in the USM group compared with that in the FSM group. Concentrations of air pollutants, such as NO, NO_2_, NO_x_, SO_2_, CO, PM_2.5_, and PM_10_ in the forest environment were significantly lower compared with those in the outdoor urban environment. Working hours were longer in the FSM group; however, the work stress evaluation as assessed by the job content questionnaire revealed no significant differences between FSM and USM. HRQOL evaluated by the World Health Organization Quality of Life-BREF questionnaire showed FSM had better HRQOL scores in the physical health domain.

**Conclusions:**

This study provides evidence of the potential beneficial effects of forest environments on CVDs and HRQOL.

## Introduction

Cardiovascular diseases (CVDs), including coronary heart disease, strokes, heart failure, hypertension, rheumatic heart disease, myocardial infarction, cardiomyopathy, and other heart diseases, are the leading causes of morbidity and mortality worldwide [1]. In particular, the increasing rate of CVDs morbidity and mortality has globally become a major focus of public health policies and epidemiological studies. The causes of CVDs are very complex; consistent evidence from both epidemiological and experimental studies have demonstrated that environmental pollution, genetics, dietary habits, diabetes, hypertension, smoking habits, and psychosocial factors increase the risk of developing CVDs [2]–[8]. In particular, environmental pollution is a major factor affecting CVDs; a large number of studies have confirmed the association of air pollution with human diseases, which is not confined to illness and also involves a higher impact on CVDs morbidity and mortality [4]. We have provided evidence that exposure to air pollution may decrease pulse pressure in the general population [9]. Moreover, in middle-aged adults, the maximum intima-media thickness (IMT) of the carotid artery is associated with the individual’s exposure to air pollution of PM_10_, PM_2.5_ absorbent, NO_2_, and NO_x_ as derived from long-term air pollution exposure estimated by land-use regression models of the European Study of Cohorts for Air Pollution Effects [10]. Ambient PM air pollution from combustion sources including elemental carbon, organic carbon, and several metals are significantly associated with cardiovascular mortality [4], [11]–[13]. Furthermore, significantly positive associations have been observed in cardiovascular hospitalizations and emergency department visits [10], [13]. Silva et al. estimated that approximately 2,100,000 premature deaths each year occur due to air pollution [14].

Forest environments are associated with positive health effects compared to urban environments. Specifically, living in a forest environment lowers cholesterol concentrations, blood pressure (BP), and pulse rates, increases levels of natural killer cell activity, has beneficial effects on cardiovascular and metabolic parameters, improves psychological wellbeing, enhances human immune function, decreases sympathetic nerve activity, and enhances parasympathetic nerve activity in the human body [15]–[19]. Experimental studies in different countries have demonstrated that forest environments can better enhance mood and work performance compared with urban environments [20], [21]. Thus, exposure to a forest environment may be therapeutic and may provide potential health benefits. Nevertheless, the studies above have investigated the effects of a forest environment on human physiological and psychological activities compared with an urban environment in experiments of short-term forest bathing trips. Therefore, studies regarding the long-term health effects for workers living in a forest environment compared with those living in an urban environment are still limited.

The objectives of this study were (1) to compare the measurements of cardiovascular parameters and health-related quality of life (HRQOL) in people working in forest and urban environments and (2) to demonstrate the forest environment has superior air quality compared with that in an urban environment.

## Materials and Methods

### Study design and population

This research has been approved by the 37th meeting (Jan. 30, 2013) of Research Ethics Committee of the National Taiwan University Hospital. The committee is organized under, and operates in accordance with, the Good Clinical Practice guidelines and governmental laws and regulations. The study participants have provided their written informed consent before received a series of detailed examinations and questionnaires.

Because the staff members in forest environment (The Experimental Forest, College of Bio-Resources and Agriculture, National Taiwan University) were 160 in total, excluding those of clinical diabetes and documented cardiovascular diseases, we determined sample size (n = 100) for healthy volunteers in forest and urban groups and sent 120 invitations to staff members for each environment for this study. All participants provided informed consent for our study before examination. Finally, the study participants included 114 urban staff members (USM) working in an urban environment in Taipei city and 107 forest staff members (FSM) working in an experimental forest of National Taiwan University, Nantou County, Taiwan. The FSM group or USM group included participants who had worked in the forest or urban environment for more than 1 year.

All participants received a series of detailed examinations and questionnaires, including baseline examination, oral glucose tolerance test (OGTT), HRQOL questionnaires, job stress assessment, cardio-ankle vascular index (CAVI), and carotid artery IMT assessment. The health examination and questionnaires (including QOL questionnaire and job stress assessments) were conducted during the initial spring season from January 31, 2013 to February 25, 2013 in Taipei and Nantou County, Taiwan.

### Site descriptions of forest and urban environments

Forest and urban environmental samples were collected from the Xitou experimental forest of National Taiwan University, and an interior office of a commercial building in Taipei, respectively. The Xitou experimental forest, located in central Taiwan, covers 2,349 ha and ranges in elevation from 500 to 2,025 m. The mean relative humidity and temperature data from 2010 to 2012 were 89% and 17°C, respectively, as indicated by the Xitou monitoring station of the Central Weather Bureau [22]. The mean annual precipitation is 2,317 mm with a distinct dry season from October to April. Our sampling site is located at an elevation of 1,150 m near the meteorological station of Xitou experimental forest (23°40′ N 120°47′ E). The commercial building where urban measurements were taken is located near the Taipei main station in Taipei city within the north of Taiwan. The mean relative humidity and temperature data were 73% and 23°C, respectively, as provided by the Taipei monitoring station of the Central Weather Bureau [22]. Interior office samples were collected in the commercial building’s 7th, 19th, and 21st floors where study subjects worked on.

### Baseline examination and OGTT

BP was measured twice after at least 5 min of rest in a sitting position. The systolic BP used in the analyses was the average of two measurements. In both groups, a standard 75-g OGTT was performed after an overnight fast of at least 10 h, with measurements from blood samples at fasting via an ante-cubital vein, and at 30 min, 1 h, 90 min, and 2 h post-challenge plasma glucose *by using Finger Stick Capillary Dried* Blood Spots. Plasma glucose and serum levels of cholesterol, triglycerides, and low- and high-density lipoprotein cholesterol (LDL-C and HDL-C) were measured using an auto-analyzer (Toshiba, TBA-200FR; Toshiba, Tokyo, Japan). Small dense LDL-C also was measured by commercial kit (Denka Seken, Tokyo, Japan).

### Exposure assessments

Instruments for forest and urban environmental monitoring were set in the Xitou experimental forest and in an interior office of a commercial building (indoor environment) in Taipei. Each monitoring system comprised a carbon monoxide analyzer (CO, Model 9841, Ecotech Inc., USA), an ozone analyzer (O_3_, TECO 49, API 400A: based on the principle of UV absorption), a nitrogen oxide analyzer (NO, NO_2_, NO_x_, Model 9841, Ecotech Inc., USA), a sulfur dioxide analyzer (SO_2_, Model 9850, Ecotech Inc., USA), a PM_10_ monitor (BAM1020, Met One Inc., Washington, USA), temperature, and relative humidity probe (Metone 083C, Met One Inc., Oregon, USA). CO, O_3_, NO, NO_2_, NO_x_, SO_2_, PM_10_, temperature, and relative humidity measurements were recorded daily every minute during the examination period. PM_2.5_ was collected by a Harvard impactor (Air Diagnostics and Engineering Inc., Maine, USA) [23]. Monitoring data of forest environment were collected by the environmental monitoring system from February 1 to 6, 2013. Indoor monitoring data of urban environment were collected by the same environmental monitoring system from February 20 to 25, 2013. Outdoor monitoring data of urban environment were provided by the Taipei air quality monitoring stations of the Taiwan Environmental Protection Administration.

### CAVI and Ankle Brachial Index (ABI)

Arterial stiffness was measured using the CAVI and ABI. CAVI is measured from the electrocardiogram, phonocardiogram, brachial artery waveform, and ankle artery waveform and was calculated by CAVI-VaSera VS-1500N (Fukuda Denshi, Tokyo, Japan). ABI and 4-limb blood pressure were also measured. The vascular screening system was placed in a quiet and independent room in the Xitou natural education area. CAVI and ABI for each participant were conducted at least twice. The test-retest reliability of CAVI and ABI in the Taiwanese population has been demonstrated as excellent [24].

### Carotid arteries IMT assessments

Carotid atherosclerosis in the common carotid artery was assessed by measuring carotid IMT, using a high-resolution B-mode, GE Vivid i ultrasound system (Horten, Norway), equipped with a 3.5–10 MHz real-time B-mode scanner. Details concerning the methods of carotid IMT measurements have been reported previously [10], [25]. In addition, a software package for vascular ultrasound was used. In general, duplex scanning refers to an ultrasound scanning procedure, recording both B-mode images of gray scale from the arteries of interest, and Doppler information about velocity and resistance in the relevant segments. The maximum and mean carotid IMT proximal to the carotid bifurcation, bulb, and internal carotid artery were measured bilaterally. CCA1 and CCA2 are points located at 0–1 cm and 1–2 cm, respectively, on CCA, distal to the carotid bifurcation. All scans were recorded on a digitalized memory system in DICOM format for subsequent off-line analysis. The carotid IMT measurement had excellent intraobserver coefficients of correlation reliability for maximum and mean carotid IMT with 0.976 and 0.988 at LCCA, and 0.970 and 0.973 at RCCA, respectively.

### Quality of life assessment questionnaire

The brief Taiwanese version of the World Health Organization Quality of Life (WHOQOL-BREF) [26] questionnaire was completed when subjects were receiving a health examination. Moreover, self-assessment of perceived health status by visual analogue scale was collected. The Taiwanese version of the WHOQOL-BREF has been demonstrated to effectively show a significant difference between normal population controls and patients in Taiwan [26], [27].

### Statistical analyses

The general characteristics, OGTT, environmental factors, working hours, and job stress scores were compared between the groups FSM and USM. Continuous variables were expressed as the mean ± standard deviation and binary variables were expressed in percentage. We used t-test to test the group mean difference of the characteristic variable if the variable is continuous and normally distributed. The Chi-square test was applied for categorical data.

Data regarding the difference between FSM and USM including CAVI, ABI, and maximum and mean values of IMT at CCA, ICA, BULB, and mean IMT (mean values of 3 sites of IMT measurements) were compared using fitted regression models with the adjustment of age, gender, fasting sugar, SBP, BMI, LDL-C, and habits of smoking and alcohol. Work stress scores and HRQOL scores also were compared between two groups in regression models with the adjustment of age, gender, fasting sugar, SBP, BMI, LDL-C, smoking, and alcohol in multiple regression models. Data analysis was performed with SAS statistical software (version 11.1, SAS Institute Inc., Cary, NC, USA).

## Results

General characteristics among participants in the FSM and USM are summarized in Table 1. The mean age of the FSM group was 44.3±10.5 years and was greater than the USM group. The FSM group had more male participants than the USM group (67.3% vs. 59.7%). The USM group had higher cholesterol and LDL-C levels than the FSM group. Coffee, tea, and drinking habits in the two groups had a significant difference. In particular, tea drinking in the FSM group (92.5%) was higher than that in the USM group (57.5%).

**Table 1:** General characteristics of urban and forest staff member groups.

	Urban	Forest	*p* value
Variables	N = 114	N = 107	
Age (years)	43.2±7.2	44.3±10.5	0.369
Male sex (%)	59.7	67.3	0.239
BMI (kg/m^2^)	24.4±3.8	24.9±3.7	0.418
Waist circumference (cm)	83.3±10.1	84.0±10.6	0.662
Systolic BP (mmHg)	115.1±15.0	119.2±16.5	0.103
Diastolic BP (mmHg)	73.1±10.7	72.9±10.9	0.659
Hypertension (%)	20.2	23.3	0.566
Hypertension with medication (%)	9.7	4.7	0.154
Cholesterol (mg/dL)	225.3±33.3	207.1±30.1	<0.001
Cholesterol ≥200 mg/dL (%)	74.6	59.8	0.019
Hyperlipidemia with medication (%)	1.8	1.9	0.949
Triglycerides (mg/dL)	135.7±111.5	140.9±110.0	0.730
HDL-C (mg/dL)	60.7±15.5	57.6±14.4	0.123
LDL-C (mg/dL)	142.2±31.9	129.7±29.0	0.003
LDL-C ≥130 mg/dL (%)	60.5	46.7	0.040
Small dense LDL-C (mg/dL)	34.6±15.6	33.8±17.3	0.724
Small dense LDL-C/LDL-C	0.24±0.1	0.26±0.1	0.279
Coffee (%)	71.7	53.9	0.007
<360 ml/day	40.7	46.2	
≥360 ml/day	31.0	7.7	
Tea (%)	57.5	92.5	<0.001
<500 ml/day	46.0	77.4	
≥500 ml/day	11.5	15.1	
Alcohol drinking (%)	20.2	41.1	<0.001
1–2 times/week	11.4	16.8	
≥3 times/week	8.8	24.3	
Smoking (%)	27.2	39.3	0.057
ex-smoker	12.3	15.9	
current	14.9	23.4	
Exercise (%)	45.9	54.4	0.216

Continuous variables were expressed as mean ± SD and *t*-test were used to make comparisons. For categorical data, *χ* ^2^ test was used.

OGTT results are presented in Table 2. Mean fasting plasma glucose in the USM group was 104.1±20.1 mg/dL and was higher than that in the FSM group (99.1±14.2 mg/dL). After OGTT, the 2-h plasma glucose concentration of the USM and FSM groups were 133.8±41.5, and 129.0±40.4 mg/dL, respectively. There was a significantly higher prevalence of impaired glucose tolerance and pre-diabetes mellitus in the USM group.

**Table 2:** Oral glucose tolerance test of urban and forest staff member groups.

	Urban	Forest	*p* value^a^
	N = 114	N = 107	
Fasting plasma glucose (mg/dL)	104.1±20.1	99.1±14.2	0.031
30 min (mg/dL)	168.4±39.1	161.7±33.9	0.177
60 min (mg/dL)	165.3±49.4	162.2±47.8	0.641
90 min (mg/dL)	149.7±45.3	142.2±46.6	0.225
120 min (mg/dL)	133.8±41.5	129.0±40.4	0.380
HbA1C (%)	5.6±0.7	5.6±0.6	0.710
OGTT Diabetes mellitus (%)	12.3	14.0	0.702
Impaired glucose tolerance (%)	26.3	11.2	0.004
Pre-diabetes mellitus (%)	19.3	6.5	0.005

a Comparisons of means between the two groups were based on t-statistic for continuous variables and Chi-square test for the categorical variables.

The working hours, job stress scores, and WHOQOL-BREF domain scores are presented in Table 3. The mean of working hours per week was significantly higher in the FSM group (51.4±11.5 h) compared to the USM group (45.5±11.1 h). No significant difference was observed between scores of job stress and work support between the two groups. Furthermore, according to mean WHOQOL-BREF domain scores, the FSM group scored higher than the USM group in all four domains. In particular, after controlling associated covariates in a regression model, the score of physical health was significantly higher in the FSM group than the USM group (29.6±3.6 vs. 29.0±4.2).

**Table 3:** Work hours, job stress scores (Job Content Questionnaires), and WHOQOL-BREF domain scores of urban and forest staff member groups.

	Urban	Forest	*p* value^a^
	N = 114	N = 107	
Work hours per week	45.5±11.1	51.4±11.5	<.0001
Job stress			
Control	25.9±3.2	25.8±3.3	0.622
Demand	15.8±2.5	15.3±2.2	0.131
Boss support	12.0±1.6	11.8±1.8	0.479
Colleague Support	12.3±1.2	12.1±1.2	0.4255
Work insecurity	14.9±2.5	14.2±3.0	0.084
Work place justice	24.1±3.1	24.6±3.5	0.2981
WHOQOL-BREF, domains			
Physical health	29.0±4.2	29.6±3.6	0.0069
Psychological	20.5±3.4	21.7±2.8	0.1656
Social relationship	13.5±2.1	14.2±2.1	0.0974
Environment	34.5±5.2	34.5±4.5	0.7888

a The test was based on regression models adjusted for age, gender, fasting sugar, systolic BP, BMI, LDL-C, smoking, and alcohol.

The mean concentration of air pollutants (SO_2_, NO, NO_2_, NO_x_, CO, O_3_, and PM_10_), temperature, and relative humidity are presented in Table 4. Considering the results of the monitoring analysis, there were significant differences in SO_2_, NO, NO_2_, NO_x_, CO, PM_10_, temperature, and relative humidity factors between the urban (indoors and outdoors) and forest environments. The forest environment had higher O_3_ concentration than the indoor urban environment, however there were no significant differences between of forest environment (23.1 ppb) and outdoor urban environments (25.4 ppb). The mean concentration of PM_2.5_ in the forest environment was lower than in the urban environment (indoor and outdoor). Despite a non-significant trend for PM_2.5_ between the forest and indoor urban environments, the mean concentration of PM_2.5_ in the outdoor urban environment was 37.2±24.0 µg/m^3^, which was higher than in the forest environment (7.2±3.9 µg/m^3^), with a significant difference (*p*<0.028).

**Table 4:** Environmental monitoring in forest and urban environments.

	Forest environment	Urban environment		*P_1_* value^a^	*P_2_* value^b^
		Indoor	Outdoor		
	N^c^ = 144	N = 144	N = 144		
SO_2_ (ppb)	2.0±0.1	3.8±1.4	2.7±1.4	<.001	<.001
NO (ppb)	2.5±1.0	20.5±20.4	6.3±6.2	<.001	<.001
NO_2_ (ppb)	3.1±1.6	10.7±2.8	25.4±11.8	<.001	<.0001
NO_x_ (ppb)	5.6±2.0	31.3±20.8	31.7±16.5	<.001	<.001
CO (ppm)	0.3±0.1	1.3±0.8	0.6±0.3	<.001	<.001
O_3_ (ppb)	23.1±13.4	1.9±0.8	25.4±16.1	<.001	0.137
Temperature (°C)	14.5±3.8	22.0±1.3	18.0±2.6	<.001	<.001
Relative humidity	87.3±12.5	55.2±3.7	78.3±9.1	<.001	<.001
PM_10_ (µg/m^3^)	20.3±9.2	15.9±4.3	48.0±29.4	<.001	<.001

a *P* ***_1_*** value corresponds to t-test on difference between the XiTou and Urban site (Indoor).

b *P* ***_2_*** value corresponds to t-test on difference between the XiTou and Wanhua site (Outdoor) of EPA, Taipei, Taiwan.

c N corresponds to the sample size of hourly average data.

The CAVI and ABI among participants of the FSM and USM groups are presented in Table 5. Although the mean CAVI values of the USM group (7.48±0.81) were slightly higher than those of the FSM group (7.41±1.01), there was no significant difference from the fitted regression models with the adjustment of age, gender, fasting sugar, systolic BP, BMI, LDL-C, smoking, and alcohol consumption. However, the mean ABI was 1.13±0.07 in the USM group, which was significantly higher than that in the FSM group (1.06±0.08). Table 6 shows the carotid IMT measurements among participants of both groups. A significantly lower mean carotid IMT at ICA was observed in the FSM group than that in the USM group. The results of maximum and mean of all carotid IMT measurements in the USM group were higher than in the FSM group.

**Table 5:** Cardio-ankle vascular index (CAVI) and ankle brachia index (ABI) of urban and forest staff member groups.

	Urban	Forest	*p* value^a^
	N = 114	N = 107	
CAVI			
Right	7.48±0.89	7.44±1.04	0.342
Left	7.47±0.76	7.37±1.00	0.177
Mean	7.48±0.81	7.41±1.01	0.242
ABI			
Right	1.129±0.08	1.061±0.08	<0.001
Left	1.131±0.08	1.061±0.08	<0.001
Mean	1.130±0.07	1.060±0.08	<0.001

a Tests of difference in each mean index between urban and forest groups using regression models with adjustment of age, gender, fasting sugar, systolic BP, BMI, LDL-C, smoking and alcohol drinking habit.

**Table 6:** Carotid intima-media thickness (IMT) of the urban and forest staff member groups.

	Urban	Forest	*p* value^a^
	N = 114	N = 107	
Carotid IMT, mm			
Common carotid artery			
Max	0.674±0.115	0.682±0.132	0.280
Mean	0.552±0.093	0.562±0.114	0.432
Internal carotid artery			
Maximum	0.597±0.095	0.582±0.104	0.066
Mean	0.498±0.076	0.481±0.083	0.033
Bulb			
Maximum	0.702±0.137	0.686±0.159	0.169
Mean	0.582±0.103	0.567±0.129	0.150
IMT maximum^b^	0.658±0.092	0.650±0.110	0.047
IMT mean^b^	0.544±0.072	0.536±0.090	0.046

a Based on regression models with the adjustment of age, gender, fasting sugar, systolic BP, BMI, LDL-C, smoking and alcohol drinking habit.

b IMT mean and maximum are the mean and maximum values of combining three sites of carotid arteries.

## Discussion

This is the first study to demonstrate the health effects of forest environment by comparing traditional cardiovascular risk factors, noninvasive cardiovascular assessments, and detailed environmental monitoring simultaneously in middle-aged workers living in forest and urban environments. Studies have demonstrated that psychosocial and environmental factors play an important role in predicting cardiovascular health [4], [20]. In this study, in addition to the higher levels of cholesterol and glucose and a prevalence of pre-diabetes, environmental factors may be accountable for the significant differences observed between the two groups. The statistically significant differences observed in this study imply that subclinical CVDs markers of carotid IMT are better in subjects working and living in a forest environment compared to those working and living in urban environments.

In middle-aged adults, the levels of blood pressure, cholesterol, fasting glucose, and carotid IMT increased with age and were higher in males [28]. The carotid IMT of the FSM group was better than those of the USM group, despite having a greater mean age and a higher percentage of males. These results provide evidence of the beneficial health effects of living in a forest environment compared to living in an urban environment. The prevalence of hypercholesterolemia and pre-diabetes in the USM group was higher than in the FSM group. Higher glucose and LDL-C levels in the blood may lead to endothelial cell lesion and damage by glycation, allowing easy access of LDL-C through arterial endothelial cells, resulting in developing CVDs with vascular stenosis and obstruction.

Urban air pollution associated with CVDs morbidity and mortality has been well documented [4], [29], [30], and the scientific statement by the American Heart Association suggested that the inhalation of air pollution may stimulate and trigger the development of atherosclerosis [4]. Our study showed a significantly lower concentration of gaseous air pollutants (i.e., NO, NO_2_, NO_x_, SO_2_, and CO) in forest environments compared to urban (indoor and outdoor) environments. In a previous study, we have proved that short-term O_3_ exposure had a significant adverse effect on aortic stiffness by CAVI measurement in young mail-carriers [31]. Since the O_3_ level in forest environment were not lower than that in urban environment, the potential hazardous effect arose from O_3_ exposure should be taken into account. This partially explains why there are no significant differences in CAVI measurement between the two groups in our study, even though all air quality indicators other than O_3_ were better in forest environment. Because CAVI measurements can be affected by short-term air pollution exposure [31], [32], the beneficial health effects of lower levels of PM and other gaseous components might be attenuated by the relative higher O_3_ concentration in forest environment. There is a need to continuously monitor the concentration of O_3_ and the changes in different seasons. As for PM_10_ and PM_2.5_, the mean concentration in the outdoor urban environment was also higher than those in the forest environment.

It is commonly recognized that PM consists of soil dust, nanoparticles, industrial emissions, sea salts, smog particles, and combustion particles from vehicle sources [20]. Within the past 10 years we have proved that air pollution in the Taipei metropolis conferred short-term adverse cardiovascular effects, such as impaired heart rate variability, impaired coagulation markers, increased inflammation indices, oxidation stress, and increased aortic stiffness, in susceptible patients as well as in healthy subjects [5], [9], [31]–[35]. Our recent study, echoing findings of studies in the US [36] and Europe [37] found that long-term residential air pollution may increase subclinical atherosclerosis indexed by carotid IMT. Furthermore, the current study confirms that a forest environment with less air pollution may benefit cardiovascular health in subclinical atherosclerosis.

The concept of QOL complements the WHO definition of health as “not only the absence of disease and infirmity but also the presence of physical, mental, and social well-being” [38]. HRQOL is the primary concern of healthcare professionals and is becoming an important health outcome indicator. Therefore, patient’s self-reported outcomes are being increasingly emphasized in recent years [39]–[45] and have become an integral component of several ongoing clinical trials [42], [43]. The Taiwanese version of WHOQOL-BREF has been demonstrated with good reliability and validity in Taiwan [26], [27], [41], [45]. In the present study, the FSM group was higher than the USM group in all four domain scores. In particular, the score of physical health (including pain, energy, sleep, mobility, activity, medication, and work) of the FSM group was significantly higher than those of the USM group. The results indicated the long-term health effects of a forest environment on CVDs as well as on HRQOL. HRQOL measurement in the USM group provides a clear understanding of the well-being of participants. In addition, viewing aggregated health and environment outcomes data helps professionals address environmental risk factors and provide beneficial health information on CVDs and HRQOL for most of the working population living in urban environments.

This study has several strengths and limitations, as follows: this is the first study to demonstrate the long-term health effects of a forest environment on subclinical CVDs and HRQOL compared with those of an urban environment. Even though the study indicated the potential beneficial health effects of living in a forest environment on CVDs and HRQOL, we indeed cannot infer the better HRQOL and subclinical CVDs in workers living in forest environment because of their better air quality by real-time monitoring of air pollutants in forest compared with those of an urban environment.

First, the CV effects of changes in seasons have not been considered in this paper. Seasonal variation of cardiovascular events may be linked to the changes of cardiovascular and endocrine/metabolic markers in different seasons [34]. However, the carotid IMT demonstrated a long-term surrogate outcome of subclinical atherosclerosis, which provides clear evidence of potential cardiovascular beneficial effects of forest environments. Second, this study included multi-discipline professionals such as healthcare providers (cardiologist, medical laboratory, and case managers), public health professionals, forest environment specialists, statisticians, and atmospheric science specialists. Because of the different occupational characteristics between the FSM and USM groups, the job content questionnaire and working conditions were compared to clarify the possible work-related factors that may bias the outcomes. Third, the beneficial health factors of a forest environment, induced by agents such as phytoncide and negative ion, have not been monitored and assessed in this paper. Information regarding the specific health benefits of organic compounds found in forest environments and on the types of mechanisms mediating the effects of phytoncide on the cardiovascular system are an important emerging area of public health and environmental sciences.

Fourth, the sample size is small, and we may not have had the statistical power to detect a significant effect on the CAVI and OGTT after 2 h post-challenge plasma glucose, and job stress with different variables. Therefore, increasing the number of samples for detailed studies of their significant differences is very important in the future.

Even though the preliminary results report the first detailed survey and environmental monitoring during early spring. The ongoing study of this project will explore the seasonal changes in the health effects of both FSM and USM groups by 4-seasons’ field environmental monitoring, and follow-up health examinations to corroborate and provide important evidence on the health effects of a natural environment as an alternative therapeutic option for CVDs.

In conclusion, this study indicated the potential health effects on subclinical marker of cardiovascular disease, in terms of CIMT and subjective HRQOL in workers living in forest environment. A large-scale and cohort study in peoples living in forest comparing to living in urban environments should be warranted.
